# Acetylation turns leucine into a drug by membrane transporter switching

**DOI:** 10.1038/s41598-021-95255-5

**Published:** 2021-08-04

**Authors:** Grant C. Churchill, Michael Strupp, Cailley Factor, Tatiana Bremova-Ertl, Mallory Factor, Marc C. Patterson, Frances M. Platt, Antony Galione

**Affiliations:** 1 Department of Pharmacology, University of Oxford, Mansfield Road, Oxford, UK; 2 Department of Neurology and German Center for Vertigo and Balance Disorders, Hospital of the Ludwig Maximilians University, Munich, Germany; 3 Department of Neurology, University Hospital Inselspital, Bern, BE Switzerland; 4 Center for Rare Diseases, University Hospital Inselspital Bern, Bern, BE Switzerland; 5 Department of Neurology, Mayo Clinic, 200 First Street SW, Rochester, MN 55905 USA

**Keywords:** Drug discovery, Medicinal chemistry, Pharmacology, Pharmacokinetics

## Abstract

Small changes to molecules can have profound effects on their pharmacological activity as exemplified by the addition of the two-carbon acetyl group to make drugs more effective by enhancing their pharmacokinetic or pharmacodynamic properties. *N*-acetyl-d,l-leucine is approved in France for vertigo and its l-enantiomer is being developed as a drug for rare and common neurological disorders. However, the precise mechanistic details of how acetylation converts leucine into a drug are unknown. Here we show that acetylation of leucine switches its uptake into cells from the l-type amino acid transporter (LAT1) used by leucine to organic anion transporters (OAT1 and OAT3) and the monocarboxylate transporter type 1 (MCT1). Both the kinetics of MCT1 (lower affinity compared to LAT1) and the ubiquitous tissue expression of MCT1 make it well suited for uptake and distribution of *N*-acetyl-l-leucine. MCT1-mediated uptake of a *N*-acetyl-l-leucine as a prodrug of leucine bypasses LAT1, the rate-limiting step in activation of leucine-mediated signalling and metabolic process inside cells such as mTOR. Converting an amino acid into an anion through acetylation reveals a way for the rational design of drugs to target anion transporters.

## Introduction

*N*-acetyl-dl-leucine has been used as an over-the-counter drug for the treatment of vertigo since 1957 (Tanganil, Laboratoires Pierre Fabre)^1^. Currently, *N*-acetyl-leucine is being intensively studied by both academia and industry as a promising treatment for several disorders with unmet medical needs including cerebellar ataxia^2–4^, cognition and mobility in the elderly^5^, lysosomal storage disorders^6–9^, migraine^10^ and restless legs syndrome^11^. Three multinational clinical trials are ongoing with the purified L-enantiomer for the treatment of Niemann-Pick disease type C, the GM2 gangliosidoses, and Ataxia-Telangiectasia^12^ (clinicaltrials.gov NCT03759639, NCT03759665, NCT03759678).

Given the promise of *N*-acetyl-l-leucine as a drug for treating many disease indications, there is intensifying interest in its mechanism of action. The current working hypothesis to reconcile the pharmacokinetic and pharmacodynamics data is that *N*-acetyl-l-leucine enters metabolic pathways, and its effects are mediated via its metabolic products^13^. Therefore, pharmacokinetic factors may be playing a major role to its mechanism of action and efficacy as a drug. Moreover, our recent findings that the enantiomers of *N*-acetyl-leucine (*N*-acetyl-l-leucine and *N*-acetyl-d-leucine) show unexpected and large differences in pharmacokinetics suggesting the involvement of differential binding sites provided by enzymes and transporters^13^.

**Figure 1:**
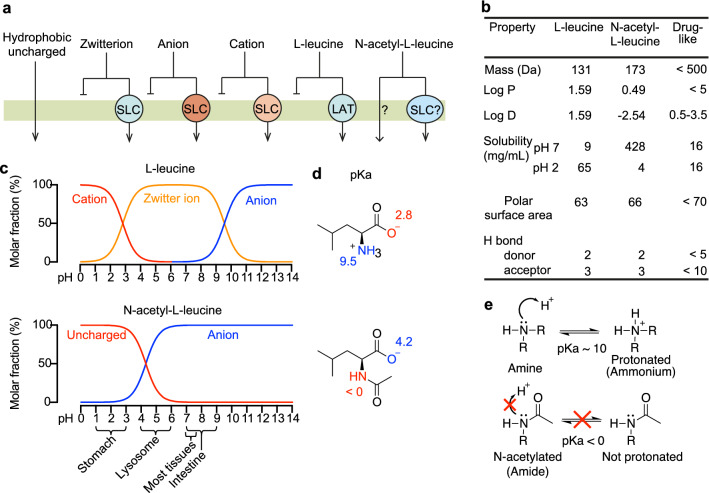
The effects of *N*-acetylation on the chemical properties and pharmacological consequences of the drug *N*-acetyl-l-leucine. (**a**) Mechanisms of absorption illustrated by crossing a membrane by passive diffusion or carrier-mediated uptake. In general, hydrophobic uncharged molecules can cross lipid bilayer membranes through simple passive diffusion, whereas charged molecules including zwitterions (a positive and negative charge within the same molecule), anions and cations cannot cross membranes without a transporter. Over 400 Solute Carrier (SLC) transporters are known with broad but overlapping selectivities for substrate. l-leucine as an obligate zwitterion at all biologically relevant pH values has an absolute requirement for its carrier, the high-affinity l-type amino Acid Transporter (LAT1). In contrast, *N*-acetyl-l-leucine can exist as a neutral species and passively cross membranes at low pH, or as an anion recognized by other transporters. (**b**) Comparison of the physicochemical properties of l-leucine and *N*-acetyl-l-leucine relating to oral bioavailability. (**c**) Speciation curves for the protonation states of l-leucine and *N*-acetyl-l-leucine. The gross charge distribution of a molecule as a function of pH is calculated as well. The dominant species is indicated in several tissues relevant to drug absorption and distribution. (**d**) Chemical structures showing charge at pH 7 with the pK_a_ of the amino and carboxylic acid groups labelled. (**e**) Lewis structures illustrating the effect of *N*-acetylation on the pK_a_ of nitrogen atom. Resonance delocalization of the lone pair electrons in the amide greatly decreases the basicity of the nitrogen relative to the amine, making the molecule neutral or charged, respectively.

Transporters exist in the membrane of all cells and are required for the uptake of small-molecule (100–500 Da) drugs that are not sufficiently hydrophobic to cross the membrane by simple passive diffusion^14,15^. As the major barrier to membrane crossing is the hydrophobic interior^16^, small, hydrophobic, neutral molecules can pass by passive diffusion (Fig. 1a), whereas hydrophilic molecules such as cations, anions and zwitterions, (including all α-amino acids) can only cross with the aid or solute carriers (SLC) transporters (Fig. 1a). Approximately 450 transporter-like genes are expressed in humans and are categorized into two major superfamilies: the solute carrier (SLC) and ATP-binding cassette (ABC) transporters^15^.

As transporters are often a rate-limiting step in drug absorption and distribution^14,15^, the objective of the present study was to explore uptake of *N*-acetyl-l-leucine. The strategy we used was to first analyze the physicochemical properties associated with good oral bioavailable drugs^17^ and how these are altered by *N*-acetylation of l-leucine. Our calculations show that at physiological pH, l-leucine is a zwitterion, whereas *N*-acetyl-l-leucine is present as mainly an anion. We then tested candidate organic anion transporters and found that *N*-acetyl-l-leucine is a translocated substrate for organic anion transporters OAT1 (SLC22A6) and OAT3 (SLC22A8) and the monocarboxylate transporter (MCT1; SLC16A1). These results provide a mechanistic explanation for why *N*-acetyl-l-leucine acts as a drug and its parent l-leucine is not.

## Results

### Acetylation alters physicochemical properties that alter membrane permeability

Several physicochemical parameters have been reported to correlate with the ability of small molecule drugs to cross membranes^17–22^. These physicochemical parameters relate to whether a small molecule can cross a membrane by passive diffusion, known in the field as being ‘drug-like’, or requires carrier-mediated transport (Fig. 1b)^22^. Therefore, it is instructive to compare the amino acid l-leucine with the modified amino acid *N*-acetyl-l-leucine to help understand how *N*-acetylation converts a l-leucine into a drug. Both molecules possess similar physicochemical parameters, except for log P, log D and solubility (Fig. 1b). Log P is the octanol:water partition coefficient for the neutral form of a compound. In contrast, log D considers the ionization state of a molecule in aqueous (biological) solution resulting from basic groups gaining a proton and acidic groups losing a proton, and as such is better correlated with passive diffusion across membranes^21,22^. For *N*-acetyl-l-leucine a log D of − 2.54 (Fig. 1b) and its speciation curve (Fig. 1c) resulting from the effect of acetylation on pK_a_ (Fig. 1d) predicts a low rate of passive diffusion across membranes^16,17,19,21^ in the neutral environment of the intestine and would require a carrier, as for short-chain fatty acids^23^ and acidic drugs such as acetylsalicylic acid^24^.

### Physicochemical properties necessitate a major role for carrier-mediated uptake

Passive diffusion of *N*-acetyl-leucine could occur across membranes in acidic conditions such as the stomach (Fig. 1c), but in environments with physiological and approximately neutral pH such as the intestine and tissues to which *N*-acetyl-leucine distributes^13,25^, carrier-mediated transport is required^26^. Moreover, pharmacokinetics following oral administration in mice revealed high levels of interference between the l- and d-enantiomers of *N*-acetyl-leucine^13^ suggestive of a specific and saturable binding site and carrier-mediated uptake. Therefore, we investigated candidate transporters for *N*-acetyl-leucine. Of the 450 possible transporters^14^ we narrowed them down to plausible candidates based on the physicochemical and steric effect of acetylation (Fig. 1a–e), reported structure–activity relationship, tissue and cell expression and their kinetic parameters (low affinity)^14^.

### The leucine transporter LAT1 does not transport *N*-acetyl-l-leucine

**Figure 2:**
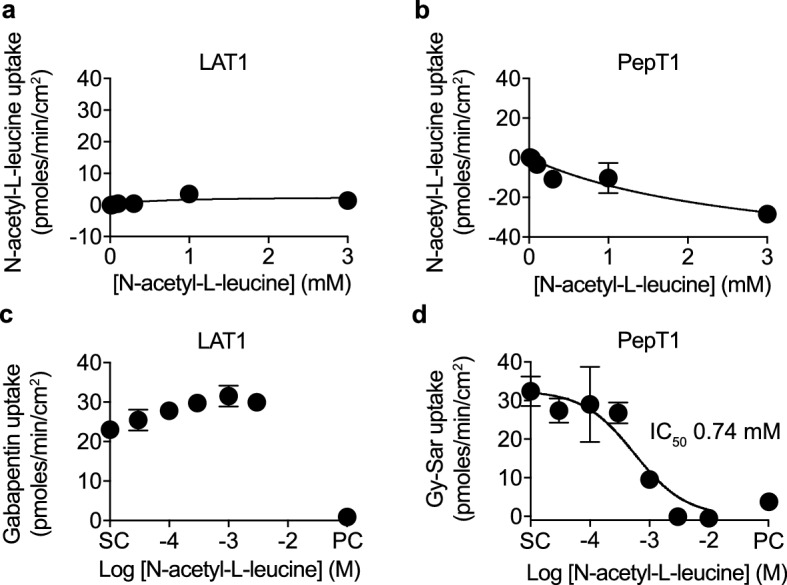
*N*-acetyl-l-leucine is not transported by either the l-type amino acid transporter (LAT1) nor the peptide transporter (PepT1). Concentration–response curves for the uptake of *N*-acetyl-l-leucine by (**a**) LAT1 and (**b**) PepT1. Concentration-inhibition curves for the inhibition of uptake of known substrates (**c**) gabapentin (10 µM) for LAT1 and (**d**) dipeptide Gly-Sar (50 µM) for PepT1. DMSO (0.5%) was the solvent control (SC) and the known inhibitor for the positive control (PC) was JPH203 (10 µM) for LAT1 and losartan (200 µM) for PepT1. Data were fit to either the Michaelis–Menten equation for uptake or the Hill equation for inhibition using the solvent control to define the top and the positive control inhibitor to define the bottom. Symbols represent the mean ± SEM, n = 3. When the error bars are smaller than the symbol, they are not visible.

We explored LAT1 (SLC7A5) as a candidate for *N*-acetyl-l-leucine transport as it is the main endogenous transporter for leucine in most cells^27,28^, 8 of the 9 essential amino acids and cysteine, as well as the amino acid-related drugs T3, T4, l-dopa, baclofen, melphalan, gabapentin and the dopamine precursor l-DOPA^29,30^. Further, LAT1 is well-characterized in terms of transport mechanism, substrate specificity and regulation^29,31^, is ubiquitously expressed in all tissues^32^ and is the rate-limiting step in leucine activation of mTORC1^33,34^, which is responsible of cell growth and survival^35^, and could explain mechanistic pharmacology of *N*-acetyl-l-leucine^2–8,10^. However, we found that *N*-acetyl-leucine was not a substrate (Fig. 2a) nor an inhibitor of LAT1 (Fig. 2c), consistent with a previous study that used an indirect assay with radioactive leucine as a surrogate^36^. Small molecules can act as inhibitors if they interact with the substrate binding site without being translocated across the membrane^26^. This a component in the kinetics of transport, where binding precedes the conformational change that catalyses translocation of the substrate across the membrane.

### The peptide transporter PepT1 does not transport *N*-acetyl-l-leucine

We next focused in on the effect of introducing an amide bond by acetylation (Fig. 1d), which sterically resembles the backbone of a di-peptide, an important recognition feature for peptide transporters. PepT1 (SLC15A1) was considered as a good transporter candidate based on it being well characterized, low affinity, and a popular target for the delivery of peptide-like prodrugs with an amino acid-like moiety designed to improve oral absorption and bioavailability of peptide-like drugs and prodrugs^14,37–40^. However, *N*-acetyl-l-leucine was not a substrate (Fig. 2b) but instead was an inhibitor of the peptide transporter PepT1, with an IC_50_ of 0.74 mM (Fig. 2d). The reason for negative values for *N*-acetyl-l-leucine uptake are unknown, but could be due to *N*-acetyl-l-leucine existing endogenously as a metabolite in cells, consistent with it being reported as a product of normal intermediary metabolism^41^, and could be resolved with stable or radiolabelled isotope uptake experiments. As most substrate-like inhibitors bind to the same binding site the inhibition is competitive and given that di-and tripeptides are present in the gut at high millimolar concentrations^28^, this level of inhibition would not be of pharmacological consequence as dietary peptides would outcompete *N*-acetyl-l-leucine at PepT1.

### The organic anion transporter OAT transports *N*-acetyl-l-leucine

**Figure 3:**
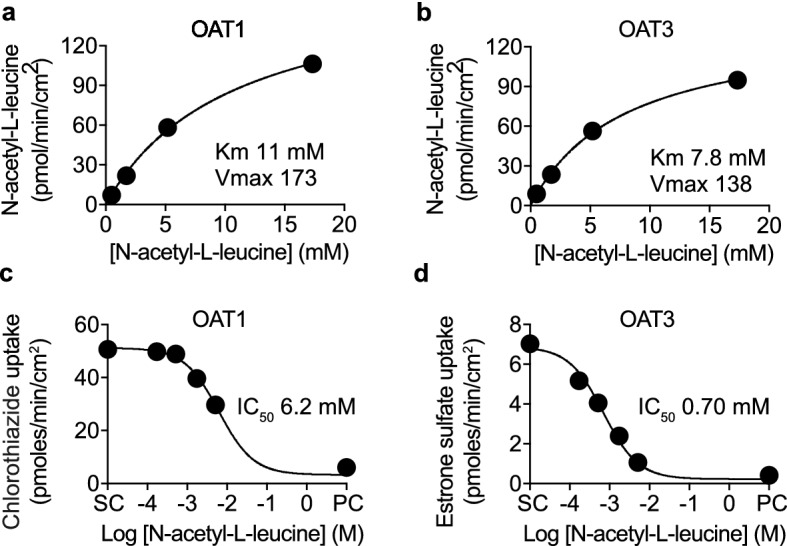
*N*-acetyl-l-leucine is transported by organic anion transporters (OAT). Concentration–response curves for the uptake of *N*-acetyl-l-leucine by (**a**) OAT1 and (**b**) OAT3. Concentration-inhibition curves for the inhibition of uptake of known substrates (**c**) chlorothiazide for OAT1 (3 µM) and (**d**) estrone-3-sulfate for OAT3 (2 µM). DMSO (0.5%) was the solvent control (SC) and the known inhibitor diclofenac (100 µM) was the positive control (PC). Data were fit to either the Michalis-Menten equation for uptake or the Hill equation for inhibition using the solvent control to define the top and the positive control inhibitor to define the bottom. Symbols represent the mean ± SEM, n = 3. When the error bars are smaller than the symbol, they are not visible.

We then pursued the hypothesis that the salient chemical feature for carrier-mediated uptake was conversion of leucine from a zwitterion to an anion (Fig. 1c). Anion transporters are known to be of relevance in drug development for their roles in drug distribution, elimination and drug-drug interactions^15,24^. We considered members of the organic anion transporters (OATs) family as candidates based on their structure–activity relationships, kinetics, pattern of tissue expression and role in the pharmacokinetics of many drugs^42–44^. *N*-acetyl-l-leucine was a substrate of OAT1 (Fig. 3a) and OAT3 (Fig. 3b). Based on the ability of *N*-acetyl-l-leucine to inhibit the uptake of the known substrates chlorothiazide and estrone sulfate (Fig. 3c,d), it inhibited OAT1 with a IC_50_ of 6.2 mM (Fig. 3c) and OAT3 with an IC_50_ of 0.70 mM (Fig. 3d). The plots in Fig. 3 represent uptake by the overexpressed transporter because they are the difference between uptake in transporter-expressing cells and non-expressing cells at a given time point (Supplementary Fig. S1). Consistent with *N*-acetyl-l-leucine being a substrate, meaning the molecule had to bind before being transported, and that transport is less efficient than binding as the IC_50_ is less than the K_m_.

### The monocarboxylate transporter MCT1 transports *N*-acetyl-l-leucine

We then reasoned that a major transporter for *N*-acetyl-l-leucine would recognize organic anions but should also be widely distributed in tissues and be accepted to have the right cellular directionality of transport to take up rather than excrete *N*-acetyl-l-leucine. One family of transporters stood out as a possibility was the proton-linked MCT family with 14 members, of which MCT1–MCT4 are well characterized^14,45^. MCT members are endogenously involved in the bidirectional movement (into and out of cells) of metabolites that perform signalling and energy/metabolic roles including ketone bodies and pyruvate, and uptake of small organic aliphatic acids produced by microbes from the gastrointestinal tract^45^. MCT family members play an essential role in the metabolism and pH regulation of cells by moving lactate into and out of cells^45^. As these metabolites are present in the micromolar to millimolar range, the kinetics of these transporter feature low affinity^45^. MCT family members are widely expressed at various tissues, including the intestine, brain, kidney and liver^46^, delivering various substrates, and a potential target for oral drug delivery as it possesses high transport capacity^47,48^. MCT1 (SLC16A1) is present in almost all tissues and is involved in several drugs and nutrients including salicylate, valproate, atorvastatin and γ-hydroxybutyrate, for both uptake and crossing the blood–brain barrier^45^.

**Figure 4:**
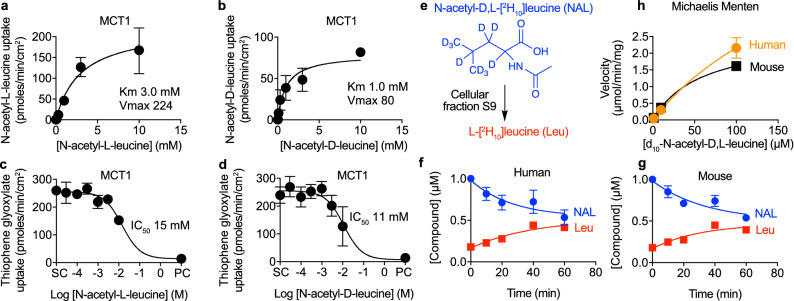
Both enantiomers of *N*-acetyl-leucine are transported by the monocarboxylate transporter (MCT1) but only the l-enantiomer is metabolized. Concentration–response curves for the uptake of (**a**) *N*-acetyl-l-leucine and (**b**) *N*-acetyl-d-leucine. Concentration-inhibition curves for the inhibition of uptake of the known substrate (**c**, **d**) 2-thiophene glyoxylate (500 µM). DMSO (0.5%) was the solvent control (SC) and the known inhibitor phloretin (500 µM) was the positive control (PC). (**e**) Chemical structure of deuterated *N*-acetyl-d,l-leucine incubated with cellular fraction S9 from liver to determine metabolism using liquid chromatography and mass spectrometry. (**f**, **g**) Time courses for loss of deuterated *N*-acetyl-d,l-leucine (1 µM initial concentration) and appearance of deuterated L-leucine for extracts derived from human and mouse livers. Data are colour-coded according to the chemical structures and names shown in **e** with deuterated *N*-acetyl-d,l-leucine in blue and deuterated l-leucine in red. (**h**) Concentration versus initial velocities relationship for metabolism yielded a K_m_ values of 216 and 69 µM and Vmax values of 6.8 and 2.6 µmol/min/mg protein for human and mouse, respectively. Data were fit to the Michalis–Menten equation for transporter uptake and metabolism or the Hill equation for transporter inhibition using the solvent control to define the top and the positive control inhibitor to define the bottom. Symbols represent the mean ± SEM, n = 3. When the error bars are smaller than the symbol, they are not visible.

We found that *N*-acetyl-l-leucine was a substrate of MCT1 with a K_m_ of 3.0 mM (Fig. 4a) and an inhibitor of MCT1 (Fig. 4c) with an IC_50_ of 15 mM. As *N*-acetyl-leucine exhibits stereospecific effects for both its pharmacodynamics^2–8,10^ and pharmacokinetics^13^, we also explored transport of the D-enantiomer. N-acetyl-D-leucine was also a substrate, with K_m_ of 1.0 mM (Fig. 4b), and an inhibitor of MCT1 with an IC_50_ of 11 mM (Fig. 4d). As both transport and inhibition require a binding step, a K_m_ higher than the IC_50_ can be attributable to the slower kinetics of translocation, but the reverse order seemed odd. We rationalized this by assuming competitive inhibition at a common binding site and then taking into account the interaction between concentration and affinity as described mechanistically by the Cheng–Prusoff equation^49^, which yielded an affinity (K_i_) of 4.3 mM for the l-enantiomer and 1.6 mM for the d-enantiomer, similar to the respective K_m_ values. This enantiomeric selectivity during transport by MCT1 provides a mechanistic explanation for the lower bioavailability of the l-enantiomer when administered as a racemate which impacts its therapeutic efficacy^13^.

### *N*-acetyl-l-leucine is metabolized to l-leucine inside cells

As enantiomeric effects on pharmacokinetics^13^ could also be due to metabolism, we monitored the metabolism of a deuterated *N*-acetyl-dl-leucine incubated with S9 microsome fraction from liver (Fig. 4e) using liquid chromatography and mass spectroscopy. The time course revealed that the disappearance of 1 µM *N*-acetyl-dl-[^2^H_10_]leucine asymptotically approached 50% of the initial concentration (i.e., 0.5 µM) and correlated well with the appearance of l-leucine for both human (Fig. 4f) and mouse (Fig. 4g) fractions. Kinetic data fit to the Michaelis–Menten equation yielded a K_m_ values of 216 and 69 µM and Vmax values of 6.8 and 2.6 µmol/min/mg protein for human and mouse, respectively (Fig. 4h). Asymmetric metabolism of the l-enantiomer relative to the d-enantiomer is consistent with previous reports of deacetylation of l but not d amino acids^50–54^. These metabolic results provide a mechanistic explanation for the faster clearance the l-enantiomer than the d-enantiomer after oral dosing of mice^13^, and are consistent with *N*-acetyl-l-leucine acting as prodrug, such as l-dopa, in which both uptake and metabolism are crucial components of its mechanism of action^55^.

## Discussion

Based on previous studies and our current results, we propose a mechanism of action of *N*-acetyl-leucine in which it is taken up and distributed by anion transporters, primarily MCT1. From the l-enantiomer, the l-leucine will be expected to be mostly utilized in the cell that deacetylates it, based studies on the uptake and utilization of ^14^C-l-leucine^56^, with the important exception of the liver, which in contrast to all other amino acids does not metabolize leucine, but rather secretes it into the circulation for the use by other tissues, particularly skeletal muscle and the central nervous system^57^. MCT1-mediated uptake of *N*-acetyl-l-leucine provides a way to bypass the easily saturable uptake via LAT1 to deliver more leucine to tissues. Indeed, at the blood–brain barrier, LAT1 is not just limited by a low K_m_ and saturation but also by competition by neural amino acids^58^, which has implications for the availability of amino acids to the central nervous system that are precursors for neurotransmitters. In contrast to the LAT1 system that is saturated by amino acids at normal blood levels (total ~ 2.4 mM)^59^, MCT1 can deliver leucine via the prodrug form *N*-acetyl-l-leucine without interference from, and disruption to, the uptake of other essential amino acids. Another aspect of transport by MCT1 is that for uptake by LAT1, leucine competes with other amino acids such as tyrosine and phenylalanine, which are precursors of neurotransmitters, and is thought to underlie the leucine toxicity in Maple Syrup Disease^60^. MCT1 provides a mechanism to signal via leucine by bypassing the normal uptake by LAT1, which is limited by glutamine in an amino acid-exchange model, and activate intracellular leucine sensors and activate powerful processes such as mTORC1^33,60^. All uptake via MCT1-mediated uptake provides a mechanistic explanation for the apparent paradox in which *N*-acetyl-l-leucine is a prodrug of leucine but leucine per se is considerably efficacious in several neurological disorders^8,57,61,62^.

The kinetics of a transporter having high or low affinity has implications for exploiting transporters for the delivery of prodrugs^48^. In general, the affinities of transporters are considered to be of high affinity when K_m_ < 0.5 mM, medium affinity when K_m_ 0.5–5 mM and low affinity when K_m_ 5–15 mM^63^. The Km for *N*-acetyl-leucine for MCT1 and the OATs places it in the medium affinity category, but it is in the range for other monocarboxylate metabolites such as pyruvate, lactate, acetoacetate, beta-hydroxybutyrate, which are in the 1–10 mM range^45,64^. The biochemical and evolutionary advantages and disadvantages of high affinity compared to low affinity have been the subject of intense theoretical and experimental studies, with one view being that they favour scarce or plentiful nutrients, respectively^65^. Far less attention has been given to drug transport, but the consequences can be profound in terms of how easily transporters are saturated and becoming limiting for the delivery of a compound, as will be discussed below.

The transport of *N*-acetyl-l-leucine by MCT1, OAT1 and OAT3 impacts on its pharmacokinetics. The lower affinity of MCT1 for *N*-acetyl-l-leucine relative to LAT1 for l-leucine prevents transporter saturation and enables operation in the linear region of the concentration-uptake curve. This can be illustrated by a numerical example comparing K_m_ and compound concentrations for l-leucine and *N*-acetyl-leucine. Taking stomach volume of a fasted individual as the accepted standard of 0.25 L^66^ a 4-g dose would result in 122 mM l-leucine and 92 mM *N*-acetyl-l-leucine. For l-leucine the LAT1 transporter would be 50% saturated at 0.2 mM (its Km), 90% saturated at 2 mM and 99% saturated at 20 mM, corresponding to oral ingestion of 0.000656 g, 0.00656 g and 0.656 g, respectively. In contrast, *N*-acetyl-l-leucine, if transported by MCT1 with a K_m_ of 3 mM it would be 50% saturated at 3 mM (its K_m_), 90% saturated at 30 mM and 99% saturated at 300 mM, corresponding to an oral ingestion of 0.149 g, 1.49 g and 14.3 g, respectively. OAT1 and OAT3 are involved mostly in drug and nutrient distribution rather than uptake from the gastrointestinal tract^67^. Moreover, based on their being primarily localized in the kidney and choroid plexus for the secretion of anionic waste and xenobiotics from the cerebrospinal fluid to the blood and urine^67^, the impact of OAT1 and OAT3 on *N*-acetyl-l-leucine would be removal from the body. However, given their higher K_m_ values of 11 mM and 7.8 mM, respectively, OAT1 and OAT3 removal would be less effective than MCT1 uptake from the blood where *N*-acetyl-l-leucine levels peak in the 0.1–1 mM range^13,68^. The relative role of these competing organic anion transporters requires further experimentation.

The broader implication of transport via MCT1 relates to its ability to transport in two different modes. MCT1, like other MCT family members, can operate unidirectionally by taking a monocarboxylate and a proton across a membrane^45^, or bidirectionally in an antiport exchange mode where monocarboxylates are swapped, which is much faster than the unidirectional mode^29,46^. MCT1 catalyses either net transport of one monocarboxylate with one proton or the exchange of one carboxylate for another^45^. MCT1 can function as a symporter or co-transporter with exchanger functionality (downhill transport) and also perform tertiary active transport (uphill transport) in the presence of either a pH gradient or when functioning as an exchanger in an antiport mode if the co-transported substrate has a membrane gradient^29,46^.

The exchange mode of MCT1 transport has important implications for both the rare disorders for which it has been demonstrated to be an effective treatment and those under current study^2–4^. One of the common metabolic changes in lysosomal storage disorders such as Nieman-Pick disease type C is a metabolic shift toward the glycolytic pathway resulting in decrease in the efficiency of ATP synthesis and an increase in the metabolic monocarboxylate end product lactate^8,69^. Cells with high concentrations of lactate would be predicted to increase the uptake of *N*-acetyl-leucine through exchange mode, thereby delivering more drug to cells with metabolic dysfunction, and simultaneously lower intracellular lactate concentrations. Increased glycolysis and lactate production is also a feature in several neuropathological disorders including Parkinson’s, Alzheimer’s, Huntington’s^70–72^ as well as in stroke and traumatic brain injury^73^. Moreover, cerebral ischemia can upregulate the expression of MCT1 in astrocytes^47,74^, again favouring delivery of drug into these cells. MCT1 provides a target for a unifying working hypothesis for how *N*-acetyl-l-leucine is effective in treating both rare and common diseases. The most wide-spread implication of this work is that it demonstrates the potential importance of organic anion families of transporters in drug mechanism of action^47,48^, in particular prodrugs created by acetylation which become acidic.

## Methods

### General chemicals

High pressure liquid chromatography (HPLC) grade methanol and acetonitrile were obtained from Merck (Darmstadt, Germany), and formic acid, acetic acid and ammonium formate were obtained from BDH Laboratory Supplies (Poole, UK). *N*-Acetyl-l-Leucine was obtained from Laboratories Pierre Fabre and dissolved directly to incubation buffer at the highest incubation concentration on the day of incubations. *N*-Acetyl-d-Leucine and all other chemicals were obtained from Sigma Aldrich (Helsinki, Finland) at the highest purity available. Water was in-house freshly prepared with a Direct-Q3 (Millipore Oy, Espoo, Finland) purification system and UP grade (ultra-pure, 18.2 MΩ).

### Human solute carrier (SLC) transporter-mediated cellular uptake and inhibition

Human OAT1 (SLC22A6) and OAT3 (SLC22A8) overexpressing HEK-293 cells and control cells without transfected transporter (Corning TransportoCellsTM) were plated to 24-well plates and cellular uptake of 10 to 100 μg/mL *N*-acetyl-l-leucine was measured in the absence and presence of transporter inhibitors. *N*-acetyl-l-leucine uptake into control cells was measured only without chemical inhibitors. Positive control substrates were incubated in parallel to demonstrate presence of active transport in each transporter transfected cell line. Cells were grown in DMEM (Gibco 4196, high glucose, without sodium pyruvate) supplemented with MEM non-essential amino acids and 10% fetal bovine serum. Cells were re-fed with fresh medium after attachment (4–6 h post-seeding). Cell were plated at a density of 4 × 10^5^/well in 24-well plates coated with poly-d-lysine. Transporter assays were conducted in 400 µL of HBSS supplemented with 10 mM Hepes, pH 7.4

Transport studies with human PepT1 (SLC15A1), LAT1 (SLC7A5) and MCT1 (SLC16A1) were conducted in MDCK-II cells expressing the human transporter or control cells expressing a vector with Green Fluorescence Protein. Cells were transfected using a proprietary, transient transfection system (OPTI-EXPRESSION Technology, BioIVT). The results from the control cells were used to correct for substrate permeation by routes other than the transporter being investigated in the study. That is, identical transport studies were conducted using cells expressing the transporter of interest and control cells which do not express the transporter, with the difference in uptake used to quantify transporter-mediated uptake. Specifically, Net Transporter Mediated Substrate Uptake (pmol/min/cm^2^) = (Cellular accumulation in the presence of the transporter) − (Mean cellular accumulation in the absence of the transporter). MDCK-II cells were maintained in DMEM with low glucose and 10% FBS. Cells were used at passages up to 40 were seeded at 60,000 ± 10,000 cells/well on 96-well plates approximately 24 h before transfection, and transport assays were performed approximately 48 h after transfection. Transport studies in MDCK-II cells were conducted in 96-well insert plates with permeable membrane (0.4 µm) and receiver tray (Millipore MultiScreen Filter 96-well).

Transport studies with PepT1 were conducted in 96-well cell culture plate with wells containing a monolayer of MDCK-II cells grown on a permeable support and a corresponding 96-well receiver tray. Cell plates are maintained at 37 °C in 5% CO_2_ atmosphere prior to initiation of the transport experiment. Pre-incubation was conducted in HBSS, pH 7.4. The transport experiments were conducted in HBSS with Bis–Tris, pH 5.5, without phenol red. Both pre-incubation and incubation were conducted at 37 °C. All final assay solutions of probe substrate, reference inhibitor or test article contain 0.5% (v/v) DMSO (vehicle control). The culture plate insert (basal side) was washed with warm HBSS three times and then blotted dry. The culture plate wells (apical side) were washed with warm HBSS three times. 100 µL of 37 °C HBSS pH 7.4 pre-incubation buffer to the apical compartment as follows: for the probe substrate transport assay, the HBSS contains vehicle; for the reference inhibition assay, the HBSS contains the reference inhibitor at the required concentration; for the test article assays, the HBSS in each well contains test compound at the specified concentration. The plates were incubated at 37 °C with orbital shaking at approximately 60 rpm for the pre-incubation (30 min), aspirated and then 100 µL of the experimental buffer was added. For the transport assays the experimental buffer was HBSS with Bis–Tris, pH 5.5 with probe substrate at the required concentration and vehicle control (0.5% DMSO); reference inhibitor and the probe substrate at the required concentrations for the reference inhibition assay; and the test compound and the probe substrate at the required concentrations for the test compound assays. Assays were incubated at 37 °C with orbital shaking at ~ 60 rpm for 5 min. Then both the apical and the basal side of the permeable support were washed four times with ice-cold PBS. Cells were extracted by adding 60 µL acetronitrile:water (50:50) to each well followed by agitation on an orbital shaker at ~ 120 rpm for 15 min. Then a 30-µL sample was taken and mixed with 200 µL of scintillation fluid and scintillation counted (1450 Microbeta, Perkin-Elmer).

Transport studies with MCT1 were performed as described for PepT1 with the following modifications. Both the pre-incubation and uptake incubation were at ambient temperatures and the assay time was 1 min. To the 30-µL extracted sample 30 µL internal standard solution (200 nM carbutamide) to the extracted sample, and then frozen until analysis by Liquid Chromatography Mass Spectrometry (LC/MS/MS). Transport studies with LAT1 were performed as described for PepT1 except that the experimental solutions were added to the basal compartment and the buffer was always HBSS pH 7.4.

The known probe substrates used as positive controls were 3 μM chlorothiazide for OAT1, 2 μM estrone-3-sulfate for OAT3, 10 µM [^3^H]gabapentin for LAT1, 50 µM [^3^H]Gly-Sar for PepT1, 500 µM thiophene-2-glyoxylic acid for MCT1. The known inhibitors used as controls for each transporter were 100 μM diclofenac for OAT1 and OAT3, 10 µM JPH203 for LAT1 and 200 µM losartan for PepT1. All incubations were in triplicate and contained 1% (OAT1 and OAT3) or 0.5% DMSO (LAT, PepT1 and MCT1). The duration of uptake was 5 min for OAT1 and OAT3, 3 min for LAT1 and PepT1 and 1 min for MCT1. Assays were performed at 37 °C with no shaking, except MCT1 at room temperature. To determine uptake due to the overexpressed transporter per se, uptake was taken as the difference between accumulation between transfected and nontransfected cells in parallel experiments at the same concentration and time. To terminate uptake the plate was placed on ice and cells washed twice with ice-cold transport buffer. To collect cells, they were detached with trypsin for 5 min, and samples of cell suspension transferred into an equal volume of ice-cold acetonitrile. Samples were stored at − 20 °C until analysis. In preparation for analysis, samples were centrifuged 20 min (4000 rpm) to separate the precipitated protein. Samples of supernatant were diluted 1:4 with phosphate buffered saline (OAT1, OAT3). Samples diluted with phosphate buffered saline were used for analysis of N-acetyl-L-leucine.

### *N*-acetyl-leucine metabolism

*N*-acetyl-d,l-[^2^H_10_]leucine was obtained from ChiroBlock (Andresenstraße, Germany) and l-[^2^H_10_]leucine was obtained from Cambridge Isotope Laboratories (Leicestershire, UK). *N*-acetyl-d,l-[^2^H_10_]leucine was incubated at 1 µM, 10 µM and 100 µM with pooled liver S9 fractions from human (mixed gender) and mouse (CD1) at 1.5 mg protein/mL in 300 µL buffer containing phosphate 100 mM, MgCl_2_ 2 mM, pH 7.4 at 37 °C for 0, 10, 20, 40, 60 min. Reactions were terminated by addition of twofold volume of 75% acetonitrile and metabolites separated by liquid chromatography and quantified by mass spectroscopy using multiple reaction monitoring (Thermo Vantage UHPLC + Thermo TSQ Quantis triple quadrupole MS Waters HSS T3 (2.1 × 100 mm, 1.8 µm column with guard filter).

### Liquid chromatography-mass spectrometry

*N*-acetyl-l-leucine, rosuvastatin, estrone-3-sulfate and chlorothiazide were separated and quantified using a Thermo Vanquish UPLC + Thermo Quantis triple quadrupole MS on a Waters Acquity HSS T3 (2.1 × 50 mm, 1.7 μm) column with guard filter. A sample of 4 µL was injected and compounds were eluted at 35 °C with a flow of 0.65 mL/min using a gradient of solvent A = 0.1% formic acid and solvent B = acetonitrile as follows (Time, A%): 0.0, 95; 0.5, 95; 2.5, 40; 3.5, 5; 4.5, 95.

### Calculations

The IC_50_ value for the test item was determined by fitting the Hill equation in the following form:

$$ A\% = \frac{Top - Bottom}{{1 + 10^{{Log[I] - LogIC_{50} }} }} + Bottom $$

where *A%* is the percent activity remaining (the mean cellular uptake observed in the solvent control sample set to 100% and the mean cellular uptake observed in the presence of the positive control inhibitor set to 0%), *Top* and *Bottom* are the upper and lower plateau of A%. *[I]* is the inhibitor concentration and IC_50_ is the inhibitor concentration where the remaining activity is at the midpoint between the *Top* and *Bottom.* To obtain robust IC_50_ fit with four test concentrations the *Top* and the *Bottom* levels were constrained to 100% and 0%, respectively. The solvent control and positive control inhibitors were also used in the curve fitting to the Hill equation as recommended as the most robust method when the test substance (N-acetyl-L-leucine in our case) could not be used at a sufficiently high concentration to provide complete inhibition^75^. In inhibition experiments, the IC_50_ is not equivalent to affinity because it is dependent on the concentrations of the substrate, the inhibitor and affinity; therefore, we assumed competitive inhibition, and calculated affinity of inhibition (K_i_) with the Cheng-Prusoff equation^49^.

Enzyme kinetic data for *N*-acetyl-l-leucine uptake and metabolism was analysed by fitting the Michaelis–Menten equation to the data. V_0_ = Vmax. [S]/K_m_ + [S], where V_0_ is initial velocity, [S] is substrate concentration, Vmax is maximum velocity and K_m_ is substrate concentration at half Vmax. All fitting was performed using GraphPad Prism 8.4 software (GraphPad Software Inc). No weighting scheme was applied.

### In silico chemical calculations

The physicochemical properties, pKa and the pH-dependent speciation curves for *N*-acetyl-l-leucine were performed with the online computational chemistry package Chemicalize (ChemAxon. https://chemicalize.com/app/calculation).

## Supplementary Information

Supplementary Figure S1.

## Data Availability

All data generated or analysed during this study are included in this published article (and its Supplementary Information files).
