# Supplementary material for: Acetylation turns leucine into a drug by membrane transporter switching
Source: Sci Rep. 2021 Aug 4;11:15812. doi: 10.1038/s41598-021-95255-5 (PMC8338929; doi:10.1038/s41598-021-95255-5)
Supplement: Supplementary file 1 — Supplementary Figure S1. [file 41598_2021_95255_MOESM1_ESM.docx]

Supplementary Information

**Acetylation turns leucine into a drug by membrane transporter switching**

Grant C. Churchill^1*^, Michael Strupp^2^, Cailley Factor^1^, Tatiana Bremova-Ertl^3,4^, Mallory Factor^1^, Marc C Patterson^5^, Frances M. Platt^1^ and Antony Galione^1^


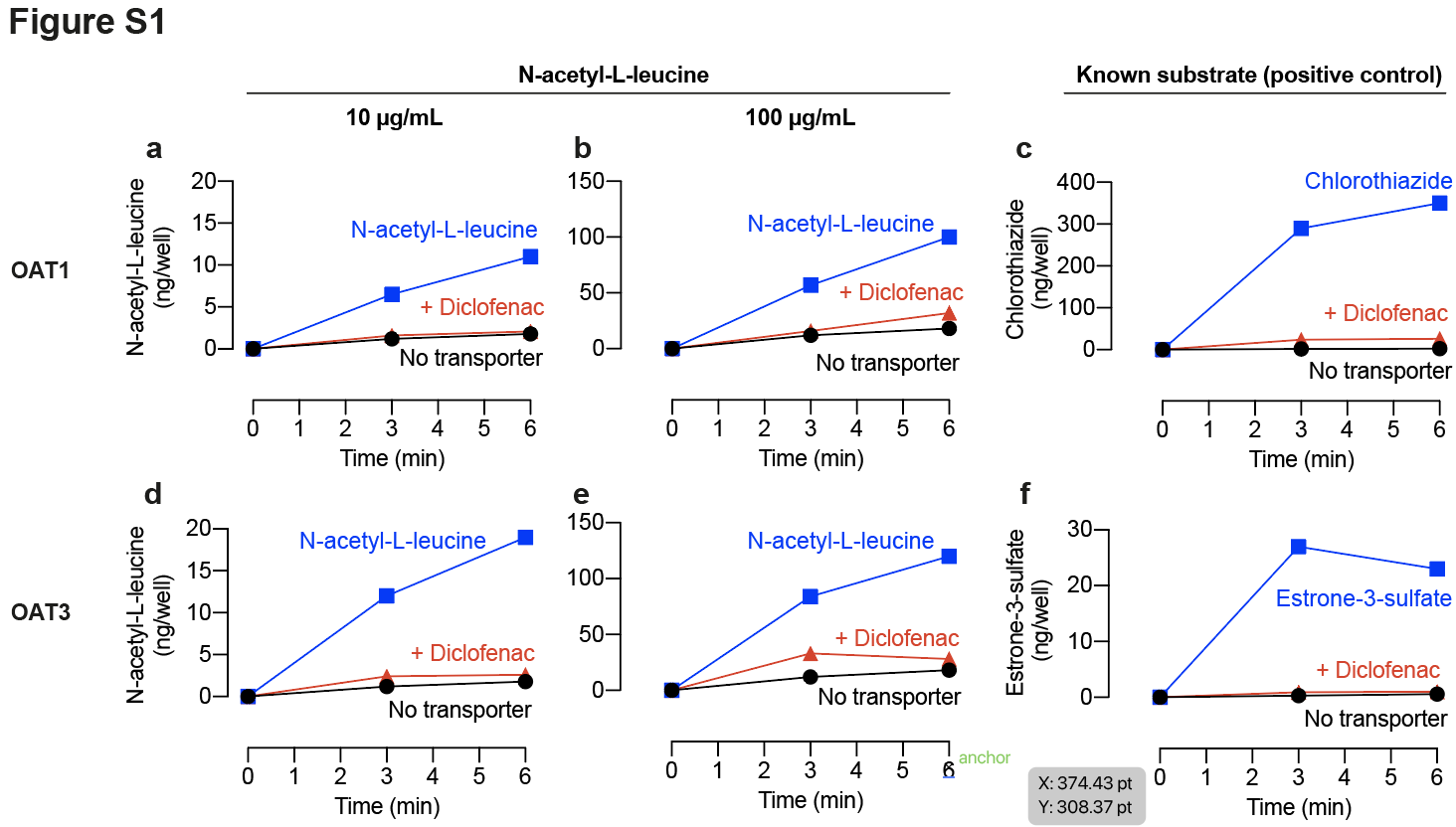


**Figure S1**. Time course of Solute Carrier (SLC) transporter-mediated active uptake of N-acetyl-L-leucine in vitro. (**a-f**) Plots N-acetyl-L-leucine or known substate uptake over time by cells expressing OAT1 (SLC22A6), OAT3 (SLC22A8) or OCT2 (SLC22A2). N-acetyl-L-leucine was present at 10 µg/mL or 100 µg/mL. A known substrate of each transporter was used as a positive control. Details of each experiment are shown as labelled on each panel. Symbols represent the mean ± SEM, n = 3. When the error bars are smaller than the symbol, they are not visible.
